# Supplementary material for: The Role of Perioperative Interleukin-6 Serum Levels on Liver Dysfunction and Infectious Complications After Hepatectomy—A Systematic Review
Source: Cancers (Basel). 2025 Sep 25;17(19):3120. doi: 10.3390/cancers17193120 (PMC12524086; doi:10.3390/cancers17193120)
Supplement: Supplementary file 1 [file cancers-17-03120-s001.zip › cancers-3868191-supplementary.pdf]

Supplementary figures:

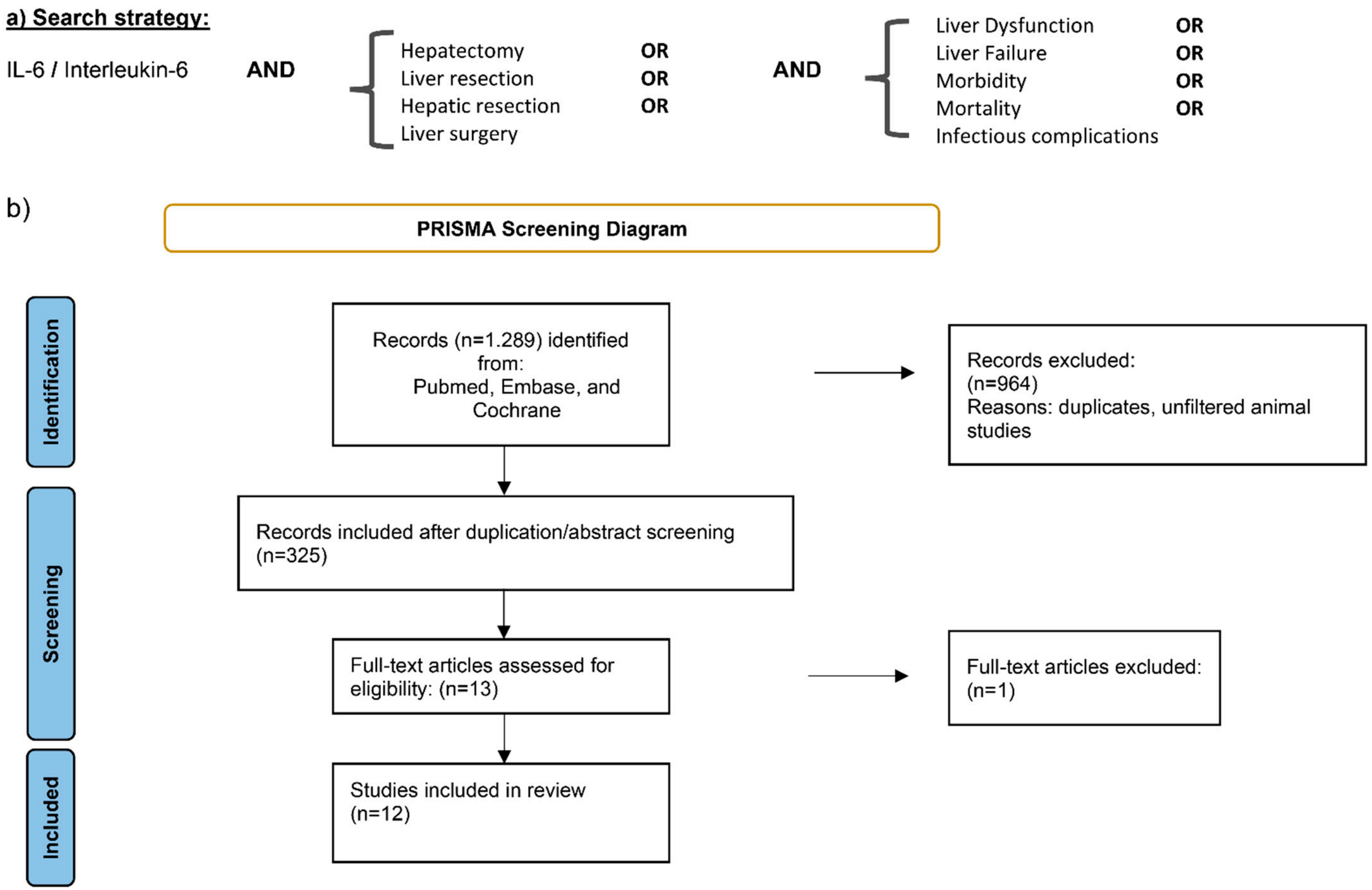

Figure S1. Literature search strategy and selection of results. A) search criteria strategy with MESH terms applied for systematic review; B) selection of results presented via Preferred Reporting Items for Systematic Reviews and Meta-Analyses (PRISMA) diagram

Supplementary tables:

Study	Oxford LOE	Described as randomized*	Described as double blind*	Description of withdrawals and dropouts*	Randomization method described and appropriate**	Double-blinding method described and appropriate**	Total points
Schwarz et al (2015)[24]	2b	1	0	1	1	0	3
Li et al (2015)[25]	2b	1	0	0	0	0	1
Sarin et al (2016)[26]	2b	1	0	0	0	0	1
Cata et al (2017)[28]	2b	1	0	0	0	0	1
Kasai et al (2017)[29]	2b	1	0	0	1	0	2

Table S1: Methodological quality of included randomized-controlled trials according to the Jadad Oxford scale.
* A study receives a score of 1 for “yes” and 0 for “no”; ** A study receives a score of 0 if no description is given, 1 if the method is described and appropriate, and -1 if the method is described but inappropriate. Overall quality is considered low for a total of 0-2 points, intermediate for 3 points, and high for 4-5 points.

**Newcastle Ottawa Quality Scale Item**	Das et al. (2001)[20]	Lan et al. (2003)[21]	Kimura et al. (2006)[22]	Strey et al. (2011)[23]	Schwarz et al. (2017)[27]	Arisaka et al. (2020)[30]	Ammann et al. (2023)[31]
**Oxford level of evidence**	2b	2b	2b	2b	2b	2b	2b
A) Selection							
Exposed truly representative of average liver surgery patient (or of subgroup)							
Selection of non-exposed from the same community							
Exposure ascertained by secure record or interview							
Demonstration of outcome of interest not present at the start of the study							
B) Comparability							
Study controls for preoperative comparability							
Study controls for operative comparability							
C) Outcome							
Adequate outcome assessment + description (IL6 - dynamics, PHLF or infectious compl.)							
Was Follow-Up Long Enough for Outcomes to Occur							
Adequacy of Follow Up of Cohorts							
**SCORE**	5	6	6	4	6	6	5

Table S2: Methodological quality of included cohort studies according to the Newcastle-Ottaway quality scale. A study with score from 7-9 has high quality, 4-6 a high risk, and 0-3 a very high risk of bias.

= consistent with Newcastle-Ottawa criteria.

**Author (year)**	**Interleukin-6 (IL-6) assay platform applied**
Das et al. (2001)	ELISA (PerSeptive Diagnostics, Cambridge, MA, USA)
Lan et al. (2003)	ELISA (Endogen, Woburn, MA, USA)
Kimura et al. (2006)	ELISA (R&D Systems Inc., Minneapolis, MN, USA)
Strey et al. (2011)	Cytometric Bead Array (Human IL-6 Flex Set and the Human Chemokine kit I, Becton-Dickinson, San Diego, CA, USA)
Schwarz et al. (2015)	RayBio® Quantibody Human Th1/Th2 Array 1 (RayBiotech, Norcross, GA, USA)
Li et al. (2015)	ELISA (Koma Biotech Inc., Seoul, Korea)
Sarin et al. (2016)	ELISA (Diaclone, Besancon, France)
Schwarz et al. (2017)	RayBio® Quantibody Human Th1/Th2 Array 1 (RayBiotech, Norcross, GA, USA)
Cata et al. (2017)	ELISA (R&D Systems Inc., Minneapolis, CA, USA and MyBioSource, Inc., San Diego, CA, USA).
Kasai et al. (2017)	ELISA (High-sensitivity Quantkine®HS, R&D Systems, Minneapolis, MN, USA)
Arisaka et al. (2020)	CLIA (two-step sandwich chemiluminescence enzyme immunoassay) – no further information provided in methods
Ammann et al. (2023)	ECLIA (Electro chemiluminescence)

Table S3: Overview of type of assay applied for assessing interleukin-6 serum levels in individual studies included. Information was derived from methods of published manuscripts or personal correspondence with main authors.
